# Waste Rubber Pyrolysis: Product Yields and Limonene Concentration

**DOI:** 10.3390/ma13194435

**Published:** 2020-10-05

**Authors:** Katarzyna Januszewicz, Paweł Kazimierski, Tomasz Suchocki, Dariusz Kardaś, Witold Lewandowski, Ewa Klugmann-Radziemska, Justyna Łuczak

**Affiliations:** 1 Department of Energy Conversion and Storage, Chemical Faculty, Gdańsk University of Technology, Narutowicza 11/12, 80-233 Gdańsk, Poland; witold.lewandowski@pg.edu.pl (W.L.); ewa.klugmann-radziemska@pg.gda.pl (E.K.-R.); 2 Institute of Fluid Flow Machinery, Polish Academy of Sciences, 80-231 Gdańsk, Poland; pawel.kazimierski@imp.gda.pl (P.K.); tsuchocki@imp.gda.pl (T.S.); dariusz.kardas@imp.gda.pl (D.K.); 3 Department of Process Engineering and Chemical Technology, Chemical Faculty, Gdańsk University of Technology, Narutowicza 11/12, 80-233 Gdańsk, Poland; justyna.luczak@pg.edu.pl

**Keywords:** limonene, waste tires, pyrolysis, waste rubber

## Abstract

Tires, conveyor belts, floor mats, and shoe soles form a main-stream of rubber waste. The amount of these used materials continuously increases due to development of the rubber market. Therefore, pro-ecological utilization (i.e., energy recycling instead of burning) and recovering valuable and recyclable materials becomes an urgent necessity. In this regard, this work was devoted to the chemical recycling of selected used rubber products, and it especially explores the possibility of limonene production. Different types of waste rubber were characterized and pyrolyzed at microgram and laboratory scales, and the results were compared. Additionally, the pyrolysis of tires, the most significant stream of rubber waste, was also conducted in a semi-technical scale reactor. The effectiveness of limonene formation in the liquid fractions obtained from different types of waste rubber was compared.

## 1. Introduction

Improvements in living standards, thus demand for elastomers, determine the continuous development of the rubber market. Just the tire market, which reached 31.5 billion dollars in 2017, is expected to increase to 41.8 billion in 2023 [1]. In this regard, waste rubber forms one of the biggest waste streams. It is mainly composed of tires, shoe soles, wiper rubber, conveyor belts, and many other products. Among these, used tires are the most prominent components of waste rubber, and their rising production is related to the dynamically developing automotive industry. Additionally, considering the well-developed mining industry in Poland, conveyor belts constitute a significant stream of rubber waste. These belts are used to transport brittle materials, such as gravel, coal, or sand. The market for conveyor belt production is developing dynamically; the expansion involves replacing multi-kilometer sections of belts.

The rubber products are composed of natural and synthetic rubber that is filled with carbon, sulphur (for the vulcanization process), zinc oxides, and oil as a plasticizer. Although the composition of these materials is characteristic for specific applications and manufacturers specifications, the products generally contain 30–50% styrene-butene rubber (SBR), 20–25% natural rubber (NR), up to 30% butyl rubber (BR), *c.a.* 30% carbon, and 1–2.5% sulphur [2]. For example, truck tires are composed of NR: 51 wt.%; SBR: 39 wt.%; and BR: 10 wt.%, whereas car tires are composed of: NR: 35 wt.% and BR: 65 wt.% [3].

The EU legislation, as well as vehicle life-cycle-end and landfill directives, forces consumers to recycle, valorise, or reuse more than 40 wt.% of tires (Directive 2008/98/EC of the European Parliament and the Council). In Poland, for example, a large amount of waste tires are co-combusted in cement plants for their high calorific value, which exceeds 30 MJ/kg [2], and the possibility to bind sulphur in the clinker [4]. Rubber waste can also be reclaimed as material in the form of fillers, granulates, or additives used for tire re-production [5]. The high calorific value of these materials provides the possibility for recovery of energy by gasification and combustion [6]. Usage of alternative energy sources, as well as different types of recycling, becomes a significant element of the product life-cycle. Moreover, waste as a source of energy constitutes an important future secondary stream of fuels. In this regard, innovations in chemical technology, involving improved reactors, catalysts, processes, and valorisation of products, are some of the main and valuable research directions.

The combustion process of waste rubber is associated with emission of harmful polycyclic aromatic hydrocarbon (PAH) and dioxins, which must be removed from the waste gas for environmental reasons. The gasification process, in turn, is related to thermal degradation of materials in a deficiency of oxygen. During this process, polymers are combusted and pyrolyzed [7]. Another important method, especially from an environmental point of view, and therefore currently intensively developed, for turning waste tires into valuable chemical compounds and fuels is pyrolysis. This is a type of thermal degradation that is also called chemical recycling. The advantage of this process is that practically all pyrolysis products (oil, carbon, and gas) can be used. Pyrolysis processes have been well described by many authors who have taken into account comparisons of different processing parameters and types of reactors that determine the yield and quality of the products [8,9]. The results obtained by Acosta et al. prove that the temperature of the process is the most important parameter. It has an influence on the composition and yield of the oil fraction. The temperature of 600 °C for the pyrolysis process confirms total decomposition of the raw material, and it results in about 6.7 wt.%. of volatile matter [10]. Recently, many intensive studies have been focused on fast pyrolysis of waste materials [11,12] as the best method of waste-free chemical recycling. Tire pyrolysis oil (TPO) has a calorific value similar to fuels and contains a series of valuable compounds, from a chemical point of view, such as limonene, PAHs, and monocyclic aromatic hydrocarbons (benzene, toluene). In this regard, it is worthwhile to use it rather than burn it [2]. A light fraction, mainly composed of volatile organic carbons, which was distilled from tire pyrolysis oil, has properties comparable to petroleum-derived gasoline and could be used as an alternative fuel [13]. The pyrolytic oil, after separation of valuable products, could also be used as a fuel component in the refinery industry. In this regard, considering the growing consumerism, it is important to develop and improve technologies that enable valorisation of the pyrolysis products [2,3].

**Figure 1:**
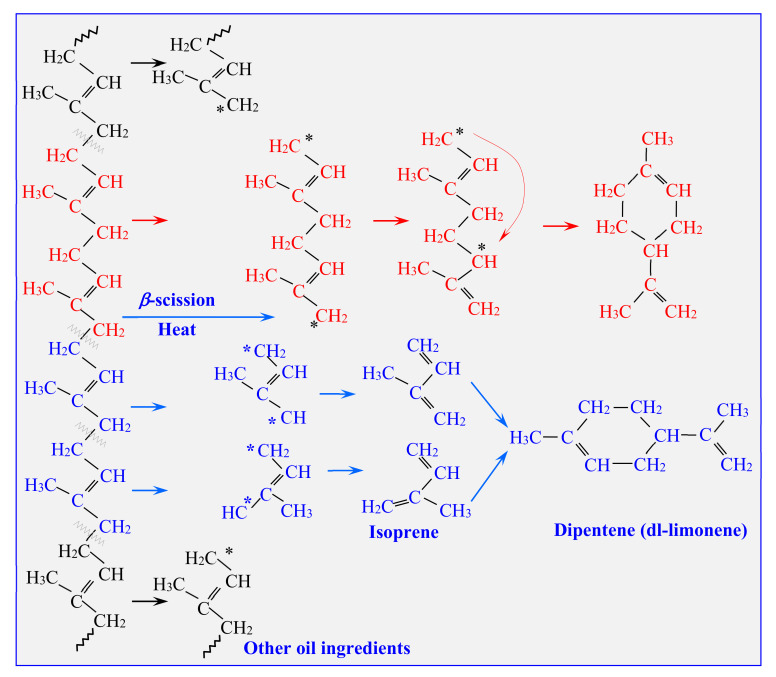
Scheme of two mechanisms for the pyrolytic decomposition of natural rubber to limonene based on [23,24,25].

Limonene (a hydrocarbon monoterpene with two double bonds, with high hydrophobicity and low polarity) is used in the industry as a solvent, an adhesive/dispersing agent for pigments in paints, an adhesive remover for cleaning purposes, and a fragrance ingredient for food and personal care products, etc. The estimated production of this compound is 50–75 million kg per year [14]. Currently, the widely used method of limonene production is extraction from citrus fruits. Alternatively, thermal depolymerisation of the natural rubber provides limonene from pyrolytic oil due to polyisoprene degradation, monomer recombination, and other reactions taking part during pyrolysis [15,16]. This mechanism is presented in Figure 1. However, a high pyrolysis temperature that is too high (above 700 °C) may result in destruction of limonene and formation of butadiene. The amount of oil and the complexity of its composition (various isomers and styrene derivatives are also formed during polymer degradation) hinder the highly efficient separation of this compound [17]. Limonene could be catalytically transformed directly into aromatic compounds, but this idea is not used on an industrial scale [18]. Alternatively, the concentration of limonene in the pyrolytic oil enables its distillation. After low-pressure fractional distillation of the oil fraction, limonene can be obtained.

Up until now, the production of limonene as a valuable component of pyrolytic oil has been analysed in a few studies in which pyrolysis of car and truck tires and latex gloves was performed [17,19,20,21,22]. Nevertheless, up until now, methods for limonene production from pyrolytic oil obtained from other waste rubber products have not been performed or compared.

In this regard, in this work, we compared the efficiency of the pyrolysis processes of four types of waste rubber streams, i.e., tires, conveyor belts, floor mats, and shoe soles. The results obtained for the mainstream of wastes (tires) in a laboratory-scale reactor were compared with pyrolysis performed in a semi-technical scale. Since composition of the substrates significantly affects the pyrolysis yield, the thermal degradation of raw materials, that are NR, SBR, and acrylonitrile-butadiene rubber (NBR), was also performed. Additionally, separation of limonene from the pyrolytic oil of the mainstream waste (tires) produced from different process scales was performed by distillation, and the limonene content in the pyrolytic oil was determined.

## 2. Experimental Part

### 2.1. Materials and Methods

The waste rubber materials (tires, conveyor belts, floor mats, and shoe soles) were received from the Polish market. Tires (a mix of car and truck tires) and sole samples were ground to a particle size distribution of 0.2–0.5 mm. Conveyor belts and floor mats were pre-washed, dried, and then shredded to 2–3 cm pieces. Considering different proportions of various types of rubber in the materials before the vulcanization and for better understanding of the pyrolysis process and the influence of rubber composition on the limonene yield, the basic components of waste rubber products, namely NR, SBR, and NBR (commercially purchased materials), were also investigated. These materials, as well as solid products of the pyrolysis, are shown in Table 1.

### 2.2. Pyrolysis Process

**Figure 2:**
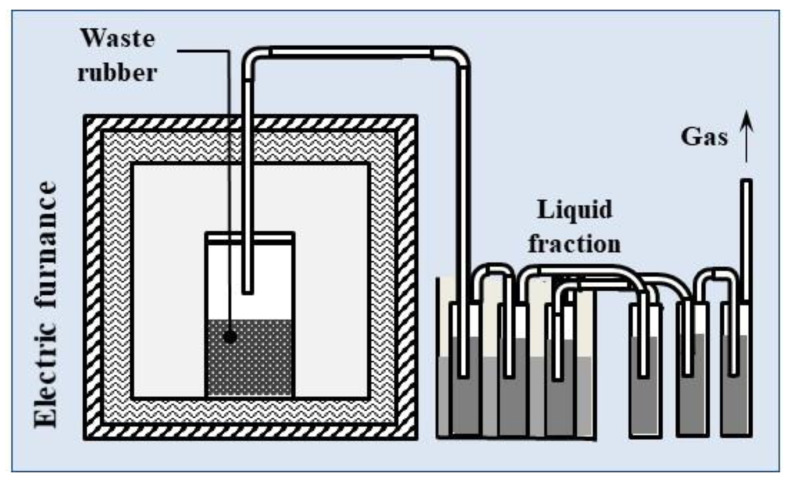
Scheme of pyrolysis laboratory-scale set-up.

The fast pyrolysis of the waste rubber samples (conveyor belts, floor mats, waste tires, soles) and the reference materials (NR, SBR, NBR) was carried out at a laboratory scale in a 250 mL steel reactor, placed in an electric muffle furnace (Figure 2). About 100 g of the sample was closed in the reactor and subjected to pyrolysis at 600 °C (heating rate was 100 °C/min) without inert gas. After completion of the process (30 min), the reactor was cooled down in an atmosphere of neutral gas in order to prevent char oxidation. The oil fraction was collected in six vessels filled with isopropanol, according to the tar protocol [26], which was developed based on a standard method for liquid fraction measurements. Three vessels were cooled using a cryostat and the next three were cooled in air. The liquid fractions were collected in the vessels and weighed. The charcoal-solid residue was also weighed, and the volatile matter was calculated by difference.

**Figure 3:**
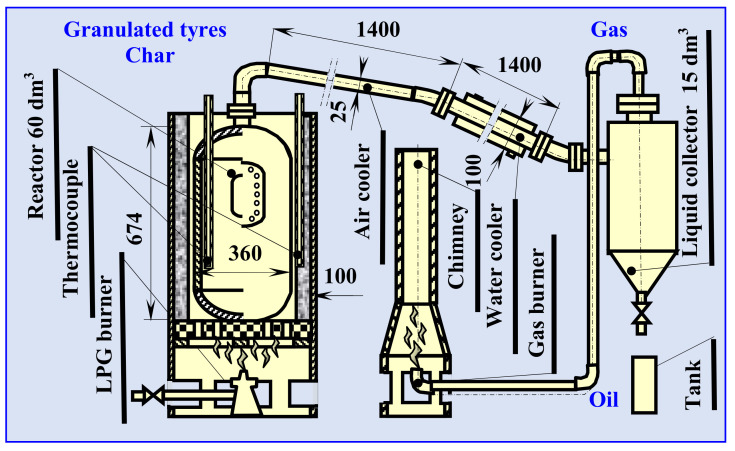
Scheme of the fixed bed reactor (semi-technical scale) used in this experimental study for granulated tire pyrolysis.

The mainstream of rubber waste (tires) was also pyrolyzed on a semi-technical scale for comparison purposes (Figure 3). A fixed bed, thermally insulated, batch reactor with diameter *D* = 0.36 m, height *H* = 0.674 m, and total volume of 60 L was used to pyrolyze tires at 450–500 °C. Due to the volume of the reactor (60 L), the slow pyrolysis process was conducted and the heating rate was 15 °C/min. The heat was provided by a tube oil burner, installed outside the furnace. The oil burner allowed the heat and exhaust gases to flow into the cuboid furnace. A feedstock of 3 kg of granulated tires was placed in the cylindrical reactor. The process temperature was measured using two thermocouples (inside the reactor and outside between the reactor and the thermal insulation). Volatiles diffused from the reactor through the air and water coolers into a 15-L liquid collector, forming the oil fraction. The gas fraction was burned, and the solid residue was discharged from the reactor.

In addition, a microgram scale pyrolysis in a thermogravimetric analyser (TGA, SDT Q600, TA Instruments, New Castle, DE, USA) was also performed. The experiments were carried out in a nitrogen atmosphere; 6 mg samples were used and the heating rate was 20 °C/min up to 750 °C. In this case, fast pyrolysis (the same as in the laboratory scale) was performed on the samples.

### 2.3. Analytical Methods

The crude oil from the semi-technical scale process was distilled and divided into four fractions: up to 160 °C, between 106–170 °C, 170–190 °C, and 190–220 °C. The boiling point of limonene is 176 °C, thus the boiling point of the four fractions, which were obtained during distillation, were near this value. All oil fractions were then analysed for limonene content by using the gas chromatography–mass spectrometry (Schimadzu-GC-2010, Schimadzu, Kioto, Japan) method of standard addition. A Rtx-5MS (60 m × 0.25 mm, 0.25 µm, Restek, Belfonte, PA, USA) was used. Helium gas was used as the carrier gas at a flow rate of 1.0 mL/min, and the sample inlet temperature was set at 300 °C. The temperature gradient was as follows: 50 °C for 4 min, raised to 320 °C at 20 °C/min, held for 15 min. The mass spectrometer source temperature was set at 210 °C.

The solid products of the pyrolysis were analysed using normal standardized methods: moisture (CEN/TS 15414-1:2010; PN-EN 15414-3:2011), volatile matter (PN-EN 15402:2011), ash (PN-EN 15403:2011). Moisture content was determined by a MAC moisture analyser (Radwag). The elemental analysis of raw materials and char were conducted using a CHNS-O analyser Flash 2000 (Thermo Scientific, Waltham, MA, USA). The calorific value was analysed by the PN-ISO 1928:2002 method, using a KL-12 MN calorimeter (PRECYZJA-BIT, Bydgoszcz, Poland).

## 3. Results and Discussion

The proximate and elemental analyses provided valuable information about composition of the raw and reference materials, as well as the pyrolysis products and chars. The relevant results are shown in Table 2. The ash content determined for tires and conveyor belts was similar (about 8–9 wt.%) and much lower than in other samples. The low content of inorganic compounds was also reflected by the high amount of elemental carbon, which was 81 wt.% and 78.5 wt.% in tires and belts, respectively. In this regard, tires provided the highest amount of solid residue (~48 wt.%) and a similar amount of volatiles (43 wt.%). Conveyor belts, in turn, were transformed mainly into volatile matter (64 wt.%). Differences in the proximate analysis results between these two samples come from their composition. The conveyor belts are made not only of rubber but also polyethylene mesh, which, during thermal treatment, changes into gaseous and liquid products, providing more volatile matter. The highest ash content was observed in the floor mats, and this was followed by the shoe sole samples (45.6 and 28.6 wt.%, respectively). This may have been related to the presence of inorganic compounds, such as flame retardants in these products. Additionally, the relatively lower carbon content in soles and floor mats (55.7 wt.% and 31.6 wt.%, respectively), as revealed by the elemental analysis, also confirmed the high presence of inorganic components. Waste soles provided the highest amount of volatile matter (68.9 wt.%) and the lowest value of fixed carbon (2.2 wt.%). Sulphur was detected in all of the waste materials; it was detected in the range of 0.2 wt.% in floor mats and up to 1.9 wt.% was observed in tires. For comparison, analysis of the reference rubber samples, namely NR, SBR, and NBR, was also performed. Unlike the waste materials, these samples did not contain ash, and they were completely transformed into volatile matter at 850 °C of elemental analysis. A relatively high nitrogen content (7.6%) in NBR was related to the presence of the nitrile group in this polymer composition.

### 3.1. Pyrolysis Process on a Microgram Scale

**Figure 4:**
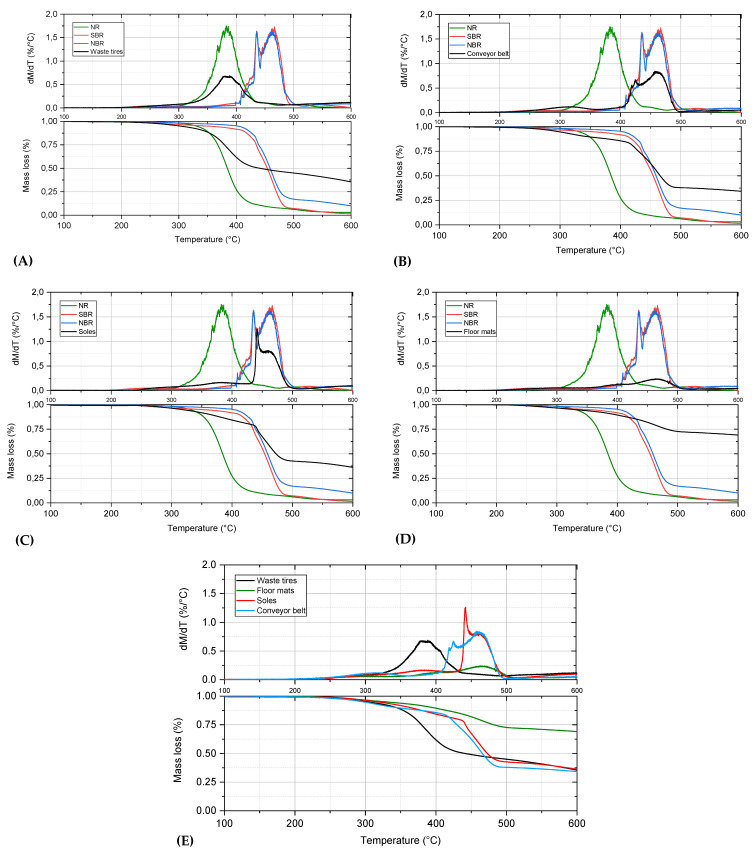
Thermogravimetric analysis (TGA) of (**A**) waste tires; (**B**) conveyor belts; (**C**) soles; (**D**) floor mats; compared with NR, NBR, and SBR degradation, and (**E**) comparison of all rubber waste.

First of all, the pyrolysis experiments of the waste rubber samples and NR, SBR, NBR reference materials were performed on a microgram scale by using thermogravimetric analysis (TGA) accompanied by derivative thermogravimetry (DTG). The reference samples were pyrolyzed for comparison and for a better understanding of the thermal degradation of waste rubber. Those materials are homogeneous with known chemical compositions, and the analyses confirmed these structures. The comparative results are presented in Figure 4.

These experiments revealed that the waste tires are composed mainly of natural rubber. The NR degradation peak corresponded well to the maximum temperature for the thermal degradation of waste tires. Conveyor belts and soles, in turn, are composed of SBR and/or NBR. The TGA characteristics of these two rubbers are too similar to differentiate which of them dominates in the waste material. The mass loss rate was similar for these samples: tires, conveyor belts, and soles. The composition of the floor mats differs from the other rubber waste. The obtained degradation temperature range, the shape of the curves, and the lowest peak height of DTG confirmed a relatively low amount of organics in the form of NBR or SBR. This observation is in agreement with the abovementioned results that showed the high content of inorganic compounds (flame retardants and rubber fillers) in this material. The lower mass loss detected for the floor mats was also reflected by the highest amount of the solid residue produced from pyrolysis. Additionally, the reason for that is involved with a different application of this product, where the high strength of the floor mats was not necessary. Therefore, in production, for economic reasons, NR, NBR, and SBR are replaced with fillers, such as carbon black [27].

The summary of the TGA results is also presented in Table 3A. The thermal degradation process was described by temperatures that are related to the 2%, 5%, 10%, and 50% mass loss by the sample. Rubber wastes were found to have lower T_2%_ decomposition temperatures than the reference materials. The decomposition temperatures ranged from 254 °C for tires to 274 °C for soles. The temperatures indicating the beginning of the degradation detected for the component polymers varied widely. The lowest was for SBR (267 °C), followed by NR (303 °C), and the highest was revealed by NBR (316 °C). A characteristic feature of the reference substances’ decomposition is the high dynamics of the process. The temperatures of 10% mass loss determined for wastes (tires, conveyor belts, and soles) were relatively similar (343–363 °C), whereas it was different for floor mats (395 °C). This was due to the larger amount of mineral fraction in this type of waste (45.6 wt.%). As a consequence, the temperature at which the sample reached 10% mass loss was elevated. This was also the reason for the highest T_50%_ temperature detected for floor mats: 763 °C. This parameter was much higher than temperatures revealed by the other rubber waste samples (441–472 °C). The average dynamics of the pyrolysis process of waste rubber samples (from 0.2%/°C for floor mats to 1.3%/°C for shoe soles) was lower than for the reference substances (1.6–1.8%/°C). During the decomposition of NR, SBR, and NBR, the maximum mass loss exceeded 1.6%/°C at 461 °C for NBR/SBR and 382 °C for NR. The relatively high dynamics of shoe sole (1.3%/°C) pyrolysis also reflects the highest amount of volatile matter (68.9 wt.%) mentioned above. The effectiveness of the rubber waste materials degradation at 750 °C was comparable (74–80 wt.%). In comparison, thermal decomposition of the reference materials in these conditions occurs almost completely, and the amount of solid residue ranges from 1.6 wt.% for SBR to 3.0 wt.% for NR. The calculation based on thermogravimetric analysis (TGA) of waste rubber sample, compared with NR, NBR, and SBR degradation, was presented in Table 3B. This analysis confirmed that the used tires contained the highest amounts of NR 26.8 wt.% and the lowest values of inorganic fraction 20.5 wt.%. Both conveyor belts and soles had similar content of NR (about 12.8 wt.%), NBR/SBR (about 30 wt.%), and inorganic fraction (about 25.2 wt.%). In comparison, the floor mats can be characterised by the highest amount of inorganic fraction and the lowest amount of NR.

### 3.2. Pyrolysis Process on a Laboratory Scale

Before undertaking a detailed analysis of the literature results on limonene production by thermal treatment of the waste materials, methods of pyrolyzing of tires were reviewed. This included pyrolysis in a vacuum, in the presence of an inert gas or catalysts, and solid–liquid pyrolysis. Nevertheless, processes are usually carried out at atmospheric pressure, in the absence of an inert gas and without a catalyst [8]. However, this was not enough to find a clear relationship between the pyrolysis scale of raw materials and the limonene yield, which is the aim of this work. A comparison of a laboratory and semi-technical scale pyrolysis of tires is important due to the excesses of this waste, as well as a scaling up point of view.

**Figure 5:**
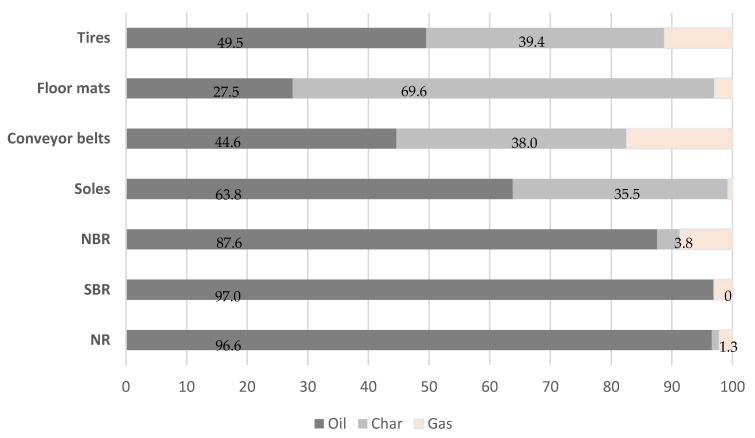
Product yields (wt.%) from the pyrolysis performed at 600 °C on a laboratory scale.

In this regard, in the next step of our investigation, the rubber waste and reference materials were subjected to fast pyrolysis on a laboratory scale, using the 250 mL pyrolytic reactor. Yields of the products from the process performed at 600 °C are shown in Figure 5.

It was observed that the pyrolysis of tires and conveyor belts provided 49.5 wt.% and 44.6 wt.% of oil and 39.4 wt.% and 38.0 wt.% of char, respectively. These results confirmed the similar quantitative composition of the rubber component mixtures revealed by TG analysis. Moreover, a lower amount of liquid fraction (27.5 wt.%) and a much higher content of solid residue (69.6 wt.%) in the floor mats confirmed the reduced content of rubber. The yields of the products obtained from shoe sole pyrolysis, especially the high amount of oil fraction (63.8 wt.%), indicated the dominant content of rubber. Higher rubber content is used for the elasticity of soles. Another component, the carbon filling that is used to increase the material strength and abrasion resistance, forms char (35.5 wt.%) [5]. Comparison of the pyrolysis yields of the abovementioned wastes with the results obtained for the three main components of all rubber products (NR, SBR, NBR) confirmed the complex composition of wastes. Thermal decomposition of the polymers provided mainly oil fractions (87.6 wt.%; 97 wt.%; and 96.6 wt.%, respectively) and small amounts or no char (0–3.8 wt.%).

The liquid fraction produced during laboratory scale pyrolysis was further analysed by gas chromatography to compare the content of limonene. The results are presented in Table 4. The highest amount of limonene (4.3 wt.%) was found in the oil fraction obtained from the pyrolysis of waste tires. This confirmed that tires (especially truck tires) contain the highest amount of natural rubber. The amount of limonene determined in the other three rubber products, floor mats, conveyor belts, and soles, was comparable: 1.6, 1.8, and 1.2 wt.%, respectively. The polyisoprene depolymerisation scheme and the mechanism of limonene formation is presented in Figure 1. The mechanism of NR monomer decomposition is related to dimerization isoprene at 450 °C and transformation into limonene. At this temperature, the composition of the pyrolytic oil fraction is rich in styrene, limonene, and monoaromatic derivatives [28]. In this work, the pyrolysis was conducted at 600 °C as a compromise between pyrolysis conditions for limonene production and completion of the thermal degradation process. Conesa et al. observed that formation of styrene up to 750 °C confirms polyisoprene cracking and a decrease in the limonene yield [28]. Considering that the highest limonene concentration in an oil fraction was obtained from tires, this material was also taken for experiments on a semi-technical scale.

### 3.3. Semi-Technical Tires Pyrolysis Process

The key aspect in designing a pyrolysis process in an industrial scale is scaling up and selection of the type of reactor, because this affects the yields of products, especially oil, and, consequently, limonene. Therefore, from all of the available reactors, as described in [8], we chose the fixed bed vertical reactor with a total volume of 60 litres (Figure 3). This pyrolizer is simple in construction and effective, and the process was conducted as slow pyrolysis. The experiment was carried out without a catalyst or an inert gas.

**Figure 6:**
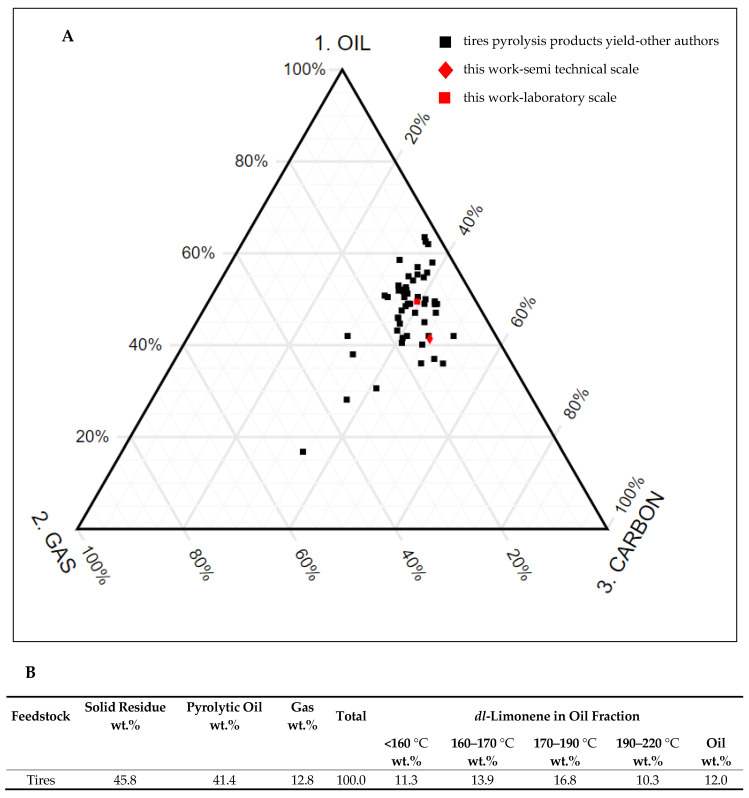
(**A**) Comparison of the results obtained in this study in the semi-technical scale (red diamond) and laboratory scale (red square), expressed as product fractions (wt.%), with the results of different authors [8] obtained by tire pyrolysis performed in different types of reactors; (**B**) The results of tire pyrolysis: product yields and limonene content in the oil fraction.

The yields of the main pyrolysis products (i.e., oil, char, and gas) were determined directly by weighing the amount of oil and the dry residue remaining in the reactor. From the thermal decomposition of about 3 kg (3,013 g) of granulated steel cordless tires, 1.25 kg of oil (41.4%) and 1.38 kg of char (45.8%) were obtained; the remaining 12.8% was gas. A comparison of our results, indicated by the red symbols, with the outcomes of other researcher’s experiments performed in fixed bed, fluidised bed, and mechanical or gravitational reactors and in different conditions [8], is presented in Figure 6A. The results of the laboratory and semi-technical scale pyrolysis obtained in this study are similar to the results from literature data [8] and the oil fraction obtained from pyrolysis of tires, performed using various reactors (Figure 6A).

The possibility of recovering limonene and other valuable aromatic compounds could increase the economic value of the whole process. Separation of limonene from the oil fraction is difficult, because it contains several compounds with similar properties and derivatives of limonene. The isolation method, which was used in this work to enrich limonene content and could be used on a technical scale, was distillation. The distillation curve of crude oil and the colour of the distilled fractions are presented in Figure S1. The limonene concentrations in the oil fraction (GC-MS analysis) and the product yields from semi-technical tire pyrolysis are shown in Figure 6A.

The pyrolytic oil obtained in the semi-technical scale (41.4 wt.%) contained much higher concentration of limonene (12.0 wt.%) in comparison to the oil from the laboratory scale process (4.3 wt.%). The reason for that was related to the parameters of the process, especially temperature and heating rate. The lower temperature and slower thermal degradation resulted in a higher yield of the liquid fraction and limonene, as well. Analogous results were obtained by Pakdel et al. In vacuum, pyrolysis performed in multiple heart furnaces and the limonene concentration in the oil fraction decreased from 23.4 wt.% at 425 °C to 5.7 wt.% at 650 °C [20]. Comparison of our results with literature data gave the impression that 41.4 wt.% of oil yielded from tires was not a high value: only two researchers using the same type of reactor obtained a lower liquid fraction yield at similar temperature (*ca* 500 °C) (Figure 6B). However, detailed analysis of the pyrolysis process conditions showed that the main reasons for the higher oil yields in these experiments was the use of an inert gas in small laboratory-scale reactors or the addition of catalysts. The high amount of solid fraction could also suggest that the pyrolysis process was not fully completed due to insufficient heating of the charge.

The highest concentration of limonene was obtained in the fraction with a boiling point between 170–190 °C (16.8 wt.%) and 160–170 °C (13.9 wt.%), which is similar to the limonene boiling point. Those fractions can be used in their existing form as valuable solvents or further processed into pure limonene. Additional values of pyrolytic oil as valuable pyrolysis products are properties, such as density, viscosity, sulphur content, and calorific value, which are similar to light fuel oil. Due to this fact, the pyrolytic liquid fraction with high yields of aromatic compounds, especially after distillation, is interesting for the refinery industry and could be used as additives to various products [29].

Another possibility for obtaining limonene could be derivatisation with methanol addition to give the mono-ether. Nevertheless, the problem with the separation of limonene ether from the other hydrocarbons (especially with those with double bonds) present in the pyrolytic oil still exists [19].

## 4. Conclusions

In this work, laboratory scale pyrolysis processes of four main rubber waste streams to form valuable products were presented. Limonene was separated by distillation of the liquid fractions. Due to the fact that the main mechanism of limonene formation is polyisoprene thermal degradation, the highest limonene content was found in the pyrolytic oil produced from waste tires. The thermogravimetric and proximate analyses of rubber waste and reference polymer samples (NR, SBR, NBR) confirmed that natural rubber was used only in waste tires. As a result of natural rubber pyrolysis, 18.4 wt.% of limonene was obtained in the oil fraction, whereas thermal treatment of waste tires provided 4.3 wt.% of this compound. The limonene concentration in the liquid fractions obtained from other rubber wastes was lower. Conducting pyrolysis of tires on a semi-technical scale resulted in a much larger amount of limonene production at 18.4 wt.%. The main reason for the larger amount of this component was lower temperature of pyrolysis, higher heating rate, and higher residence time of volatiles in the reactor.

## Figures and Tables

**Table 1:** Raw materials before and after the pyrolysis process.

**TYPE**	Natural Rubber—NR	Styrene-Butadiene Rubber—SBR	Acrylonitrile Butadiene Rubber—NBR	Conveyor Belts	Floor Mats	Waste Tires	Soles
**RAW MATERIAL**	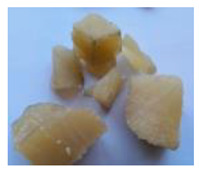	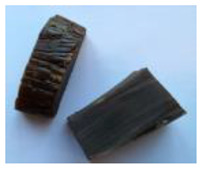	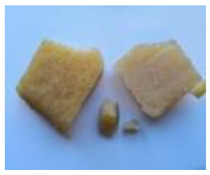	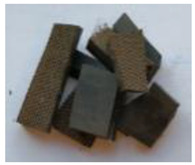	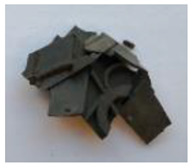	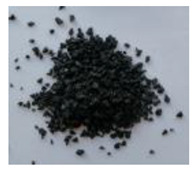	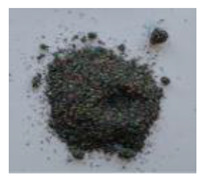
**CHAR**	without char	without char	without char	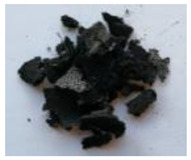	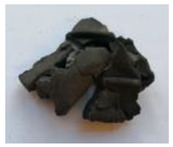	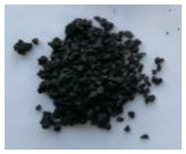	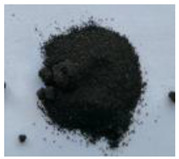

**Table 2:** Elemental composition (wt.%) and proximate analysis (wt.%) of raw materials used in the experiments, and relevant chars formed during pyrolysis.

Proximate Analysis (wt.%)				Elemental Analysis (wt.%)			
Sample	Fixed Carbon	Ash	Volatile	C	H	N	S
Tires	48.3	8.7	43.0	81.0	6.6	0.6	1.9
Char tires				65.1	0.4	0.2	0.9
Conveyor belts	28.1	7.8	64.1	78.5	6.9	0.6	0.7
Char con. belts				53.1	1.6	0.4	0.0
Floor mats	11.4	45.6	43	31.6	3.5	0.0	0.2
Char f. mats				21.4	0.2	0.0	0.0
Soles	2.2	28.6	68.9	55.7	6.9	0.6	0.7
Char soles				32.3	0.6	0.3	0.5
NR	-	0.0	100.0	85.8	11.4	1.0	0.0
NBR	-	0.0	100.0	80.6	9.9	7.6	0.0
SBR	-	0.0	100.0	85.8	10.9	0.3	0.0

**Table 3:** (**A**) Thermal decomposition characteristics of rubber waste samples pyrolyzed on a microgram scale in nitrogen atmosphere. (**B**) Composition of the elastomeric material in waste rubber, calculate based on derivative thermogravimetry (DTG).

**(A)**							
**Raw Material**	**Decomposition Temperature (C)**				**DTG Maxima**	**Char at 750 (wt.%)**	
	**T_2%_**	**T_5%_**	**T_10%_**	**T_50%_**	**T_max_ (°C)**	**(dm/dT)_max_ (%/°C)**	
Tires	254.8	302.6	343.4	441.1	381.6	0.7	20.5
Conveyor belt	256.8	296.9	343.5	465.7	459.2	0.8	26.5
Soles	274.6	317.1	363.5	472.7	441.5	1.3	23.9
Floor mats	272.6	328.2	395.5	763.5	463.4	0.2	54
NBR	315.8	401.9	421.6	458.8	461.2	1.6	1.9
SBR	267.3	350.4	411.6	453.9	461.2	1.7	1.6
NR	302.9	330.7	348.2	384.4	382.4	1.8	3
**(B)**							
**Raw Material**		**Calculate Based on DTG (wt.%)**					
		**NR**		**NBR/SBR**		**Inorganic**	
Tires		26.8		25.3		20.5	
Conveyor belt		12.7		33.4		26.5	
Soles		12.9		28.3		23.9	
Floor mats		8.4		11.9		54.0	

**Table 4:** Limonene concentration in the oil fraction (wt.%) obtained from pyrolysis of the selected raw materials.

Raw Material	Limonene wt.%
Tires	4.3
Floor mats	1.6
Conveyor belts	1.8
Soles	1.2
NBR	0.0
SBR	0.0
NR	18.4

## Supplementary Materials

The following are available online at https://www.mdpi.com/1996-1944/13/19/4435/s1, Figure S1: Distillation curve of tire pyrolysis oil. The oil fraction: a. <160 °C, b. 160–170 °C, c. 170–190 °C, 190-220 °C.
