# Supplementary material for: Waste Rubber Pyrolysis: Product Yields and Limonene Concentration
Source: Materials (Basel). 2020 Oct 5;13(19):4435. doi: 10.3390/ma13194435 (PMC7579170; doi:10.3390/ma13194435)
Supplement: Supplementary file 1 [file materials-13-04435-s001.pdf]

SUPPLMENTARY MATERIALS

Waste Rubber Pyrolysis: Product Yields and Limonene Concentration

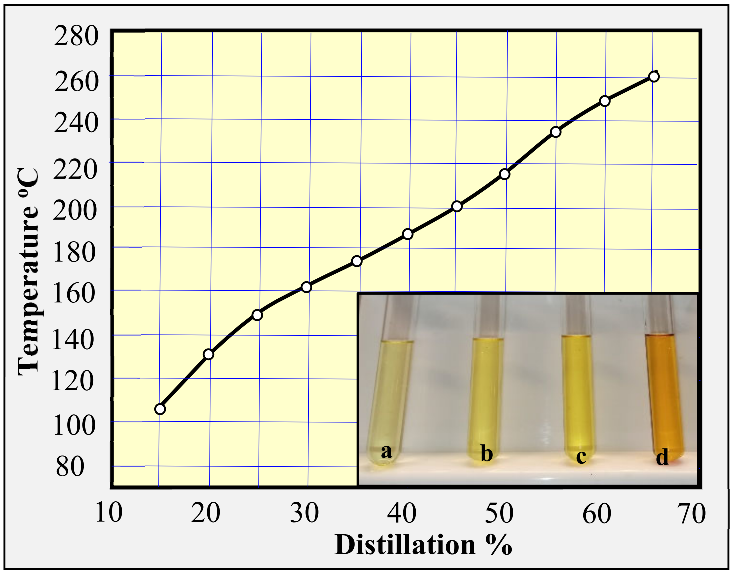

Figure S1. Distillation curve of tire pyrolysis oil. The oil fraction: **a.** <160 °C, **b.** 160–170 °C, **c.** 170–190 °C, 190–220 °C.
